# Advances in colon cancer research: *in vitro* and animal models

**DOI:** 10.1016/j.gde.2020.12.003

**Published:** 2021-02

**Authors:** Tamsin RM Lannagan, Rene Jackstadt, Simon J Leedham, Owen J Sansom

**Affiliations:** 1 Cancer Research UK Beatson Institute, Glasgow, G61 1BD, UK; 2 Heidelberg Institute for Stem Cell Technology and Experimental Medicine (HI-STEM gGmbH), Im Neuenheimer Feld 280, Heidelberg, 69120, Germany; 3 Division of Cancer Progression and Metastasis German Cancer Research Center (DKFZ), Im Neuenheimer Feld 280, Heidelberg, 69120, Germany; 4 Intestinal Stem Cell Biology Laboratory, Wellcome Trust Centre for Human Genetics, University of Oxford, Oxford, OX3 7BN, UK; 5 Institute of Cancer Sciences, University of Glasgow, Garscube Estate, Glasgow, G61 1BD, UK

## Abstract

Modelling human colon cancer has long been the ambition of researchers and oncologists with the aim to better replicate disease progression and treatment response. Advances in our understanding of genetics, stem cell biology, tumour microenvironment and immunology have prepared the groundwork for recent major advances. In the last two years the field has seen the progression of: using patient derived organoids (alone and in co-culture) as predictors of treatment response; molecular stratification of tumours that predict outcome and treatment response; mouse models of metastatic disease; and transplant models that can be used to de-risk clinical trials. We will discuss these advances in this review.

**Current Opinion in Genetics and Development** 2021, **66**:50–56

This review comes from a themed issue on **Cancer genomics**

Edited by **David J. Adams**, **Marcin Imielinski** and **C. Daniela Robles-Espinoza**

For a complete overview see the Issue and the Editorial

Available online 7th January 2021

**https://doi.org/10.1016/j.gde.2020.12.003**

0959-437X/© 2021 The Authors. Published by Elsevier Ltd. This is an open access article under the CC BY license (http://creativecommons.org/licenses/by/4.0/).

## Introduction

Colorectal cancer (CRC) with ∼800 000 deaths per year globally is still one of the most common cancers in men and women [1]. This heavy public health burden is predicted to increase if no improvement in early detection and effective interventions for late stage CRC are discovered. Currently routine colonoscopic imaging followed by surgical resection of primary tumours and oligo-metastases are the first intervention option for CRC patients [2]. Depending on stage, combination treatments of cytotoxic chemotherapies like 5-fluoruracil (5-FU), oxaliplatin or irinotecan show some benefits, which can be increased in combination with vascular endothelial growth factor receptor (VEGFR) or epidermal growth factor receptor (EGFR) treatments [3,4]. While the use of EGFR inhibitor depends on the mutation status of *KRAS* and *BRAF* [5], VEGFR inhibitors are routinely applied for stage IV patients [6]. The decisions for systemic treatments are based on routine diagnostics such as histopathological characteristics of tumours, the status of lymph-nodes and metastasis (TNM). This grading system doesn’t account for additional factors that impact on response and patients’ outcome. For example, BRAF mutations are associated with right-sided colon cancers with specific molecular features and poor-prognosis [7,8] however the clinical treatment decision is independent of these recognised characteristics. In addition, the therapies above are the same anti-proliferative combination treatments that have been used since the 1990’s, drug design and development has not kept pace with our understanding of the biology of CRC. One attempt to embrace the wide spectrum of inter-patient heterogeneity, and importantly take different molecular features into account, is the definition of molecular subtypes [9, 10, 11].

**Figure 1:**
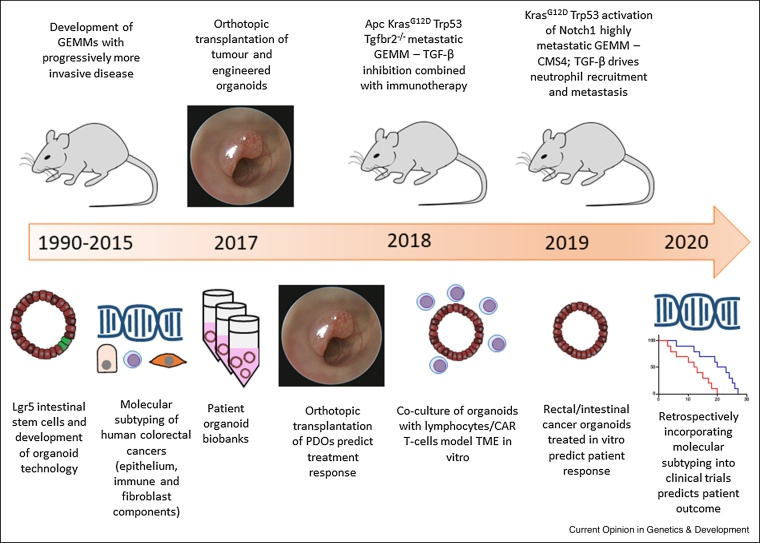
Timeline of recent advances in mouse models of CRC in parallel with molecular, technological and clinical scientific advances. Decades of research provided the field with GEMMs that permitted investigation of driver mutations in CRC, however, the models had long latency and were minimally metastatic. Concomitant discovery of intestinal stem cell markers precipitated the organoid revolution, which could be subsequently engineered using CRISPR, and provided means to store patient samples in biobanks. Molecular subtyping of CRC biopsies revealed patient stratification relating to epithelium, immune and fibroblast components. Advancements in transplantation techniques enabled orthotopic grafting of mouse or patient tumour-derived/engineered organoids, importantly PDOs were shown to be able to predict patient treatment response. 2018 and 2019 saw breakthroughs in the development of increasingly metastatic GEMMs with the KPN mouse exhibiting 100% metastasis and correlating with CMS4, the most aggressive CRC subtype. TGF-β and immunotherapy treatments demonstrated utility in reducing metastatic burden in these models. The same period of time saw advancement of *in vitro* organoid models, with both co-culture with immune cells and *in vitro* therapeutic treatment of organoids alone demonstrating patient responsiveness. More recently, the retrospective incorporation of molecular subtyping into clinical trial outcomes is predictive of patient outcome, establishing the importance of patient stratification. Modified from Figure 1 in Jackstadt and Sansom [37]. It is used under license CC BY 4.0. https://creativecommons.org/licenses/by/4.0/.

Here we discuss the most recent advances in the field of CRC research and highlight technical improvements which may lead to discovery of novel treatment concepts. Focusing particularly on the fast progressing field of organoids, the molecular characterisation of CRC, and the importance of next-generation mouse models, which aim to de-risk the translation of pre-clinical results into the clinics by improving human relevant features (Figure 1).

## Modelling CRC *in vitro* - a guide for treatments

Classical 2D adherent cell lines served for many years as valuable tools to discover key functional aspects of CRC biology; however, these cell lines show reduced complexity compared to patient tumours. Significant improvement followed identification of the intestinal stem cell (ISC) marker Lgr5 [12] and the subsequent opportunity to culture self-organising mouse intestinal organoids derived and arising from this stem cell population. The generation of mouse and human organoids from virtually every organ followed shortly [13]. Sequential genetic alterations using CRISPR/CAS9 to model acquisition of CRC driver mutations in human organoids confirmed the concept proposed by Vogelstein in 1990 [14,15]. Besides the opportunity to culture healthy tissues, tumour derived organoids were proven to retain morphological, genetic and transcriptional features of the initial founder tumour [16,17]. Importantly, cultures of CRC patient-derived organoids (PDOs) were used as avatars to predict patient response *ex vivo* [18, 19, 20, 21]. Interestingly the generation of a liver metastases PDO biobank revealed a modest inter-patient heterogeneity in pharmacological response and may serve to identify specific treatment responder versus non-responder transcriptional profiles [22]. Strikingly, treatment response following orthotopic transplantation of liver metastasis or rectal tumour PDOs into immune-deficient mice correlated with patient response [23^••^,^24^]. These elegant studies provide the proof-of-concept that tumour response and resistance can be predicted in ‘window-of-opportunity’ trials that can help guide clinical decision making. Some financial, logistical and technical limitations still present obstacles that need to be overcome before precision medicine of organoids and drug-discovery can be applied routinely in the clinic [25].

One substantial limitation of organoid cultures is the lack of tumour microenvironment (TME) components and other physiological parameters. The remarkable results for checkpoint inhibition in immunogenic microsatellite instable (MSI) colon cancer [26] demonstrates the potential power of microenvironmental epithelial cell-extrinsic influence over cancer cell fate and highlights the necessity of immune competent model systems when testing cancer therapies. To circumvent this limitation, co-culture systems of organoids with mesenchymal cells and lymphocytes have been developed [27,28]. Neal *et al.* modulated air-liquid interphases and demonstrated that lymphocytes in culture treated with PD1 or PDL1 inhibitors generate a cytotoxic response to cancer cells [27]. In co-culture systems of chimeric antigen receptor (CAR)-engineered lymphocytes with PDOs, specific cancer cell killing was demonstrated and proposed as a personalised *in vitro* testing platform [29]. These studies demonstrate that a holistic approach is a suitable way to investigate cancer cell-immune cell interactions with relevance for cancer therapies. However, the culture conditions in which these cells are propagated can never truly replicate the nuanced tissue intercompartmental signalling ecosystem within a tumour, and this should be considered when using these systems.

## Stratified medicine and tailored treatments

It is a critical challenge to distinguish responders from non-responders, or identify stage III patients with increased likelihood of recurrence. In an ideal scenario this discrimination would take place prior to treatment, consequently our understanding of the underlying molecular features that drive resistance are crucial to this endeavour. One strategy is to classify patients, and identify inter-patient heterogeneity, with the use of transcriptional profiles of whole tumours, including epithelial cancer cell and stromal components. Various studies identified 3–6 subtypes with distinct molecular features and pathway activation (reviewed in Ref. [30]). An international consortium set out to clarify these classifications and ultimately defined four comprehensive consensus molecular subtypes (CMSs) with different molecular features [9]. CMS1 is characterized by mutations in BRAF and microsatellite instability/DNA mismatch repair deficiency (MSI/dMMR). These tumours display high cytotoxic lymphocyte infiltration response to checkpoint inhibition, thus called the ‘immune’ subtype. CMS2 the ‘canonical’ subtype shows high WNT signalling activation, epithelial clusters and high levels of chromosomal instability (CIN). CMS3 differs slightly and shows increased KRAS mutations accompanied by association to metabolic changes and is accordingly the ‘metabolic’ subtype. CMS4, the ‘mesenchymal’ subtype, is dominated by stromal content, fibroblasts, monocytic and lymphocytic lineages. Molecularly, this subtype shows increased activation of TGF-β signalling, epithelial-mesenchymal transition (EMT), and predicts worst survival. Together, these classifications demonstrate heterogeneity between patients and highlight the opportunity to treat patients according to their subtype. A number of clinical trials retrospectively incorporating the CMSs show specific response [30,31,32^•^] demonstrating the importance of patient stratification and the value of implementing CMS in clinical practice. However, a number of confounding factors can influence the accuracy of CMS calling. Particularly, the composition of the stromal compartment is of increased importance in CMS1/4 tumours and contributes strongly to CMS identification. To this end, bioinformatics tools were developed to query the stromal composition in detail [33]. While this was useful to decipher the stromal content of the tumour, CMS2/3 tumours comprise of much more epithelium. To capture the signature of epithelial cells, which are influenced by the environment and account for intra-tumoral heterogeneity, the CRC cell intrinsic subtypes (CRIS) were developed [10,11]. Importantly, CRIS are less dependent on regional variation within a tumour, thus subtypes are classified much more robustly [34]. However, a faster and less costly way to identify CMS is needed to increase the use in clinical diagnosis. The use of image based CMS (imCMS), where machine learning algorithms are capable of automated detection of CMS specific morphological features from H&E slide alone, is a promising application with potential for clinical implication [35]. In addition, CMS calling has been optimized at protein level by immunohistochemistry for CDX2, FRMD6, HTR2B, pan-cytokeratin, ZEB1 and microsatellite status [36].

## *In vivo* models of intestinal cancer: emerging pre-clinical tools

As we have covered in a previous review [37], the suite of mouse models available for pre-clinical research were lacking a number of essential features relevant to human disease. In this section, we will focus on the recent improvements of genetically engineered mouse models (GEMMs) and the improved transplantation of organoids to recapitulate human colon cancer progression.

It is beyond doubt that GEMMs are the most appropriate tools to recapitulate the complexity of the tumour ecosystem [38]. Spontaneous tumours that develop in a fully immune-competent environment create systemic inflammation which substantially contributes to tumour progression [39]. They are also subject to the same evolutionary bottlenecks of transformation, invasion and metastasis as human tumours and undergo a natural selection during expansion. However, a persistent issue of these models was the low penetrance of metastasis to distant organs. If mice developed spontaneous metastasis the rate was below 25% with a latency which made pre-clinical investigations nearly impossible [37]. To this end, we developed a model of CMS4 intestinal cancer, driven by *Kras*^G12D^ mutation, which is highly penetrant to the liver (>80%) with a strong desmoplastic reaction in primary tumours and metastasis [40^••^]. In combination with *Trp53* mutation, activation of Notch1 signalling rewired the TME towards an immunosuppressive neutrophil rich environment driven by TGF-β mediated neutrophil attraction. In line with these findings, Germann *et al.* reported the importance of a neutrophil rich TME for the activation of latent TGF-β ligands by neutrophil secreted metalloproteinases [41]. Besides the generation of an immunosuppressive TME, expression of activated epithelial Notch1 (*Rosa26*^N1icd^) drives poor prognosis subtypes CMS4 and CRIS-B; however, Notch1 activation in the context of *Apc* mutations (*Apc*^fl/+^) didn’t trigger a subtype switch and tumours remained non-metastatic CMS2/3 tumours [40^••^]. Further evidence for the role of Notch signalling in metastatic CRC came from a recent publication by Varga *et al.* whom observed increased Notch3 activation in a GEMM with mutations in *Trp53* and constitutive activation of AKT [42]. The mice developed metastatic CRC (∼80% lymph nodes affected) that resembled CMS4 when treated with the carcinogen azoxymethane (AOM) and therapeutic treatment with a NOTCH3 antibody reduced primary and metastatic burden [42].

Classical CRC is thought to progress from adenoma to adenocarcinoma with initiating mutations in the APC tumour suppressor and subsequent mutations in KRAS, TP53, SMAD4. When mutations along the classical route of progression to colon cancer were combined in mice, they develop spontaneous metastatic tumours albeit with a penetrance of maximal 40% (often lower) [43, 44, 45]. Orthotopic transplantation of organoids derived from such tumours, either into the colon or the liver, has proven to be a rapid and reliable tool to investigate multiple aspects of advanced CRC [42,43,45, 46, 47, 48, 49]. For example, these novel models were used to demonstrate the effect of CXCR2 and TGF-β inhibition on metastasis [40^••^], with increased efficacy when combined with checkpoint inhibition [43,50]. Furthermore, the role of Lgr5 positive and negative cancer stem cells were explored using a transplant model with the surprising finding that only liver metastases, and not the primary tumour, show a remarkable sensitivity to Lgr5^+^ cell depletion when diphtheria toxin receptor mediated killing was utilised [49,51^•^]. Moreover, engineered organoids monitored by intra-vital imaging uncovered that most disseminated cells when they leave the primary tumour and seed to the liver are Lgr5 negative [51^•^]. During expansion to macro-metastasis these cells de-differentiate and regain Lgr5 expression. In addition, primary tumours showed recurrence post depletion [49,52], indicating a fundamental impact of the TME on stem cell plasticity. Future studies should address mechanisms, which control this plasticity and test the therapeutic potential.

Importantly, these GEMMs and organoid models were proven to recapitulate characteristics of human cancers and could be stratified into different CMS [40^••^,^43^,^44^]. It is interesting to note that the field is lacking an epithelial CMS2/3 GEMM that metastasises spontaneously with high penetrance. Half of stage IV CRC patients comprise of CMS2/3 and half of liver metastases are CMS2, thus CMS2 appears able to drive metastatic progression [53,54]. Alternatively, it may be an issue of intra-tumour heterogeneity compromising accurate subtyping and it is possible that CMS2/3 tumours simply contain a CMS4 region that drives the aggressive disease [55]. As previously discussed, CRIS analysis could be used as an alternative to CMS to more robustly classify the epithelium in tumours. Another possibility is the temporary acquisition of more aggressive features, CMS2 CRC has been demonstrated to subtype switch from CMS2 in bulk human CRC biopsies to CMS4 in cells budding from the invasive margin, suggestive of a mechanism as to how these subtypes could become metastatic[56]. As observed with the Lgr5 stem cell plasticity described above it may be that upon seeding to the distant site the established metastases revert to the CMS of the primary tumour rather than persist as CMS4. Obtaining multiple tumour biopsies from different regions and treating patients according to the most aggressive pathology contained within may improve outcomes.

**Figure 2:**
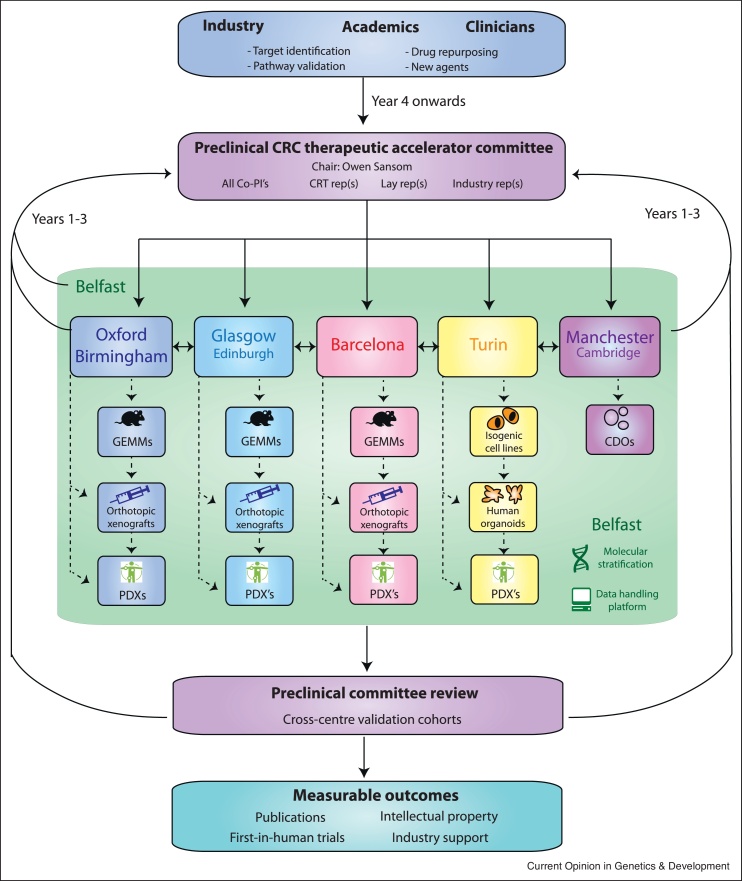
ACRCelerate: Colorectal Cancer Stratified Medicine Network Platform design and governance. Project proposals arising from industry partners, academics or clinicians will be submitted to a preclinical therapeutic committee for discussion. Execution of the project will be allocated to the various hubs based on centre expertise and interest before cross centre validation is undertaken. We also seek to develop complementary circulating tumour cell-derived organoid (CDO) technology. This pipeline will contribute to the advancement of translational research in CRC by bringing together CMS relevant GEMMs, orthotopic transplant models, PDOs and xenografts to couple preclinical information with clinical science.

It will be an important task for the future to generate a comprehensive cross-comparison of mouse models and human datasets to allow for precise subtyping of mouse models. To this end, we have initiated a Cancer Research UK funded consortium (ACRCelerate: Colorectal Cancer Stratified Medicine Network) which will characterise a suite of state-of-the-art mouse models that recapitulate human disease to generate reliable and robust pre-clinical data and de-risk the failure of promising drug candidates in clinical trials (Figure 2).

## Conclusions

Overall, the *in vitro* and *in vivo* landscape of CRC mouse models in recent years has improved substantially; however, a number of key features are not appropriately represented in the currently available models. PDO cultures have demonstrated their usefulness in correlating with patient outcome and predicting patient responsiveness to therapy. The ongoing development of co-culture techniques with TME components is essential to more closely replicate patient disease, and ultimately treatment response on a time-scale that could direct patient treatment in real-time. As discussed above, subtyping patient disease and mouse models has been proven as a valuable concept, and although mouse tumours show a narrower phenotype compared to the wide spectrum of human disease, this feature should be used to identify the most representative models of specific patient groups. As the number of mouse models expands it may be possible to take a primary approach to subtype mouse tumours themselves, before relating back to human subtypes. This may allow functional assessment of the impact of key driver genes. With the recent advantages in single cell techniques, tumour heterogeneity can be analysed with a much higher resolution. Initial comparative studies in lung cancer and CRC highlight a striking similarity between human and mouse tumours [57^•^,^58^]. Single cell technologies will allow for an even better cross-comparison of human and mouse tumours, to identify targetable modules in various cell lineages. Additionally, spontaneous GEMMs which precisely generate tumours in human relevant parts of the colon are needed. These models, driven by genetic alterations detected in humans should enable the community to investigate therapeutic options for example specifically in right-sided and rectal cancer under fully immune-competent conditions.

## Conflict of interest statement

SJL has received funding from UCB and Redx Pharma. OJS has received funding from AstraZeneca, Redx Pharma, BMS and Novartis.

## References and recommended reading

Papers of particular interest, published within the period of review, have been highlighted as:

• of special interest
•• of outstanding interest

## CRediT authorship contribution statement

**Tamsin RM Lannagan:** Writing - original draft. **Rene Jackstadt:** Writing - original draft. **Simon J Leedham:** Writing - review & editing. **Owen J Sansom:** Writing - review & editing.
